# Dual roles for immune metagenes in breast cancer prognosis and therapy prediction

**DOI:** 10.1186/s13073-014-0080-8

**Published:** 2014-10-28

**Authors:** Angela Alistar, Jeff W Chou, Srikanth Nagalla, Michael A Black, Ralph D’Agostino, Lance D Miller

**Affiliations:** Department of Internal Medicine, Wake Forest School of Medicine, Medical Center Blvd, Winston-Salem, NC 27157 USA; Department of Biostatistical Sciences, Wake Forest School of Medicine, Medical Center Blvd, Winston-Salem, NC 27157 USA; Department of Medicine, Jefferson Medical College, 1015 Chestnut Street, Suite 1321, Philadelphia, PA 19107 USA; Department of Biochemistry, Otago School of Medical Sciences, University of Otago, 710 Cumberland Street, Dunedin, 9054 New Zealand; Department of Cancer Biology, Wake Forest School of Medicine, Medical Center Blvd, Winston-Salem, NC 27157 USA; The Comprehensive Cancer Center of Wake Forest University, Medical Center Blvd, Winston Salem, NC 27157 USA

## Abstract

**Background:**

Neoadjuvant chemotherapy for breast cancer leads to considerable variability in clinical responses, with only 10 to 20% of cases achieving complete pathologic responses (pCR). Biological and clinical factors that determine the extent of pCR are incompletely understood. Mounting evidence indicates that the patient’s immune system contributes to tumor regression and can be modulated by therapies. The cell types most frequently observed with this association are effector tumor infiltrating lymphocytes (TILs), such as cytotoxic T cells, natural killer cells and B cells. We and others have shown that the relative abundance of TILs in breast cancer can be quantified by intratumoral transcript levels of coordinately expressed, immune cell-specific genes. Through expression microarray analysis, we recently discovered three immune gene signatures, or metagenes, that appear to reflect the relative abundance of distinct tumor-infiltrating leukocyte populations. The B/P (B cell/plasma cell), T/NK (T cell/natural killer cell) and M/D (monocyte/dendritic cell) immune metagenes were significantly associated with distant metastasis-free survival of patients with highly proliferative cancer of the basal-like, HER2-enriched and luminal B intrinsic subtypes.

**Methods:**

Given the histopathological evidence that TIL abundance is predictive of neoadjuvant treatment efficacy, we evaluated the therapy-predictive potential of the prognostic immune metagenes. We hypothesized that pre-chemotherapy immune gene signatures would be significantly predictive of tumor response. In a multi-institutional, meta-cohort analysis of 701 breast cancer patients receiving neoadjuvant chemotherapy, gene expression profiles of tumor biopsies were investigated by logistic regression to determine the existence of therapy-predictive interactions between the immune metagenes, tumor proliferative capacity, and intrinsic subtypes.

**Results:**

By univariate analysis, the B/P, T/NK and M/D metagenes were all significantly and positively associated with favorable pathologic responses. In multivariate analyses, proliferative capacity and intrinsic subtype altered the significance of the immune metagenes in different ways, with the M/D and B/P metagenes achieving the greatest overall significance after adjustment for other variables.

**Conclusions:**

Gene expression signatures of infiltrating immune cells carry both prognostic and therapy-predictive value that is impacted by tumor proliferative capacity and intrinsic subtype. Anti-tumor functions of plasma B cells and myeloid-derived antigen-presenting cells may explain more variability in pathologic response to neoadjuvant chemotherapy than previously recognized.

**Electronic supplementary material:**

The online version of this article (doi:10.1186/s13073-014-0080-8) contains supplementary material, which is available to authorized users.

## Background

Breast cancer is the most common cancer in women worldwide with over 200,000 new cases diagnosed in the US each year [1]. An increasing fraction of these patients are being offered systemic treatment prior to definitive surgery, known as neoadjuvant therapy. While the intent of conventional systemic therapy is to reduce the risk of distant recurrence (that is, for patients with non-metastatic invasive breast cancer), the primary objective of neoadjuvant therapy is to reduce tumor volume, thereby improving surgical outcomes for patients who desire breast conservation or for whom a primary surgical approach is otherwise not medically feasible. Moreover, according to the results of clinical trials in the US and Europe, neoadjuvant chemotherapy is as effective as adjuvant chemotherapy at prolonging patient disease-free survival, distant metastasis-free survival (DMFS) and overall survival [2,3].

Like adjuvant therapy, the current standards of care for neoadjuvant treatment include chemotherapy, endocrine therapy, and biologic therapy (for example, HER2-directed therapy). A corollary benefit of neoadjuvant treatment, however, is that it may serve as an *in vivo* chemosensitivity test, allowing for early evaluation of the efficacy of systemic therapy and the possible discontinuation of ineffective treatment [4,5]. Neoadjuvant chemotherapy can lead to significant clinical response rates of 60 to 80%, although only 10 to 20% of patients will exhibit a complete pathologic response (pCR) [2,6]. pCR is typically defined as tumor regression marked by the absence of detectable residual disease in the breast and lymph nodes at surgery. Recently, more precise diagnostic models that better quantify the extent of residual disease have been developed [7–9]. For example, measurement of residual cancer burden (RCB) provides a categorical index for tumor responsiveness to neoadjuvant treatment based on size and cellularity of the primary tumor and number and size of involved lymph nodes [9].

The biological mechanisms that influence tumor responsiveness in the neoadjuvant setting are not clearly understood. Routinely administered cytotoxic agents such as anthracyclines and taxanes are known to inhibit replication of rapidly dividing tumor cells by blocking nucleic acid synthesis, or by disrupting microtubule function, respectively. Not surprisingly, markers of tumor cell proliferation, including Ki-67 staining and histologic grade, have been observed to be significantly associated with higher rates of pCR in breast tumors [10,11]. Other therapy-predictive features of breast cancer, such as negative estrogen receptor status and HER2 overexpression, have also been identified [11–13], although not without some degree of controversy [14] and with little indication of clinically applicable predictive value. Mounting evidence now indicates that host-therapy interactions influence tumor responsiveness to neoadjuvant treatment, and that the patient’s immune system, in particular, can actively contribute to tumor regression. In mouse models of cancer, where intact tumors were treated with anthracycline-based chemotherapies, tumor regression was observed in immunocompetent mice, while the same tumors transplanted into immunodeficient mice failed to respond [15–18]. Furthermore, anthracycline-based chemotherapies have been observed to induce rapid and prominent tumor infiltration by Th1-oriented effector immune cells in mice and in some human breast cancer patients [19–21]. Indeed, the mere abundance of tumor-infiltrating leukocytes, namely CD3+/CD8+ T lymphocytes, has been robustly correlated with pCR in the neoadjuvant setting [22–25] as well as relapse-free and overall survival of breast cancer patients [25–29]. In more recent years, microarray expression profiling studies in breast and other tumor types have identified immune gene signatures from whole tumor RNA extracts that reflect the abundance of tumor-infiltrating immune cells [30–38]. We and others have found that the biological and phenotypic properties of the genes comprising these signatures implicate distinct immune cell lineages [34–37,39,40], and that combinations of these immune genes correlate with patient outcomes ranging from recurrence-free survival [30,32,36–43] to tumor regression in the neoadjuvant setting [44–47].

Recently, we reported in Nagalla *et al*. [36] the discovery of three biologically distinct immune gene signatures, or metagenes, in a large microarray dataset comprising 1,954 breast tumor expression profiles. Through gene ontology enrichment analysis and demonstration of immune cell type-specific expression patterns, we provided evidence that these immune metagenes reflect tumor-infiltrating populations of: 1) B cells/plasma B cells (B/P) marked by the high expression of IgG antibody isotype-related genes; 2) a T cell/natural killer cell-specific population (T/NK) likely reflecting a predominantly Th1-type functional orientation; and 3) a monocyte/dendritic cell population (M/D) marked by the expression of myeloid specific markers and a host of major histocompatibility complex (MHC) class II antigen-presenting molecules. Each of these signatures was found to be significantly and positively associated with DMFS of patients. This protective effect, however, was mostly restricted to highly proliferative cancers of the basal-like, HER2-enriched and luminal B (LumB) intrinsic molecular subtypes. By contrast, the same immune signatures exhibited little to no protective effect in tumors of low or intermediate proliferative potential or those classified as luminal A (LumA) or claudin-low (CL) subtypes. In the present work, we sought to evaluate the therapy-predictive potential of these immune metagenes in the context of neoadjuvant chemotherapy for breast cancer, and in the presence of other covariates such as proliferation and intrinsic molecular subtype.

## Methods

### Microarray data origination and patient characteristics

We assembled a retrospective microarray database (MDACC-701) of breast tumor expression profiles derived from five well-curated, publicly available datasets housed in the NCBI’s Gene Expression Omnibus (GEO). The dataset accession numbers are GSE25066, GSE20194, GSE20271, GSE22093 and GSE23988. Specific details of the patient cohorts are described elsewhere [42,48–51] and summarized below. All microarray experiments pertaining to these datasets were conducted at the Department of Pathology, MD Anderson Cancer Center (MDACC), Houston, Texas, as part of several international and multicenter studies conducted between 2000 and 2010. According to previously published reports [48–51] for each study the research protocol was approved by one or more institutional review boards, and all participating patients provided written informed consent consistent with the principles of the Declaration of Helsinki. Expression profiles were generated from RNA samples isolated from fine needle aspirates (FNAs) or needle core biopsies of breast tumors (stage I to III) collected prior to treatment with neoadjuvant chemotherapy. All RNA samples were analyzed on the Affymetrix U133A or U133 PLUS 2.0 GeneChip platforms. In multiple instances, a tumor expression profile was associated with more than one GEO dataset. To create MDACC-701, we downloaded a total of 1,128 tumor profiles from the five datasets then filtered for the unique (nonredundant) profiles using a custom script to measure correlations between all pair-wise combinations. In this manner, redundant profiles (that is, tumor profiles included in more than one dataset) and hybridization repeats could be identified by virtue of high (or perfect) pair-wise correlations. After consolidating the unique profiles, we further excluded a small number of outlier arrays (n = 7) based on low signal intensity distributions. Upon completion of filtering, 701 tumor expression profiles remained. Microarray probe sets were filtered to include only those common to both array platforms (22,277 probe sets). Corresponding patient and clinical characteristics were obtained from supplemental data associated with the original publications or from data associated with the GEO accessions. For redundant profiles, comparison of data entries across the different clinical data sources revealed a small number of discrepancies. In these instances, the discordant clinical data points were re-labeled as 'uncertain' and censored from our analyses. Patient and clinical characteristics of MDACC-701 are summarized in Table 1 and consolidated on a per-sample basis in Additional file 1. Of note, none of the tumor expression profiles of MDACC-701 overlap with the datasets used to discover and characterize the immune metagenes in Nagalla *et al*. [36].

**Table 1:** **Clinical characteristics of the neo-adjuvant cohort**

**Characteristic**	**MDACC-701**	
	**Number**	**Percentage**
**Age at diagnosis, years**		
≤40	134	19
41-50	247	35
>50	320	46
**Chemotherapy type**		
FAC	74	11
FAC + paclitaxel	236	34
FAC + docetaxel	61	9
FEC	33	5
FEC + paclitaxel	73	10
Paclitaxel	65	9
Docetaxel	39	6
Unspecified	120	17
**Chemotherapy response**		
pCR or RCB 0,1	188	27
No pCR or RCB 2,3	492	70
Unspecified	21	3
**Estrogen receptor status**		
Positive	385	55
Negative	300	43
Unspecified	16	2
**HER2/neu status**		
Positive	65	9
Negative	561	80
Unspecified	75	11
**Intrinsic subtypes**		
Basal	211	30
LumA	216	31
LumB	135	19
HER2-E	74	11
Claudin-low	47	7
Normal-like	16	2
Unspecified	2	0.3

FAC = cyclophosphamide, doxorubicin, 5-FU; FEC =5-FU, epirubicin, cyclophosphamide; HER2-E, HER2-enriched; LumA = luminal A; LumB = luminal B; pCR = complete pathologic response; RCB = residual cancer burden.

### Microarray data processing

The tumor expression profiles were normalized by the Robust Multi-array Average (RMA) algorithm [52] using R software and the *Affy package* library file from the Bioconductor project [53]. Normalization was performed within each dataset, and normalized expression values (log2 signal intensities) were corrected for batch effects across datasets using the COMBAT empirical Bayes method [54]. PAM50 subtypes (including the CL classification) were assigned to each sample using previously published methodology [36,55–57].

### Metagene construction

A summary of the content of the individual metagenes is as follows: proliferation (P) metagene (61 probe sets, 54 genes), B/P metagene (65 probe sets, 40 genes), T/NK metagene (52 probe sets, 46 genes) and the M/D metagene (30 probe sets, 19 genes). The proliferation and immune metagenes were constructed as described in Nagalla *et al*. [36] based on the probe set and gene name assignments listed in Additional File six of that publication. The probe sets 200904_at, 204834_at and 211742_s_at, which overlapped between the T/NK and M/D metagenes, were excluded to enhance distinction between metagenes. Metagene scores were computed for each tumor by averaging the signal intensities of the genes comprising each metagene as described previously [36]. When multiple probe sets corresponded to the same gene designation, these probe sets were averaged first, prior to cross-gene averaging of signal intensities. Tumors were grouped into metagene tertiles by ranking tumors by metagene scores and identifying the 33rd and 66th percentile thresholds. As the cellular composition of a tumor biopsy can differ depending on the tissue sampling method used [58], we sought to compare the metagene score distributions between the surgically acquired tumor specimens that we previously used to derive the metagenes (n = 1,954 tumor samples) [36] and the confirmed FNA biopsy samples that comprise the majority of the current study (n = 482 tumor samples). Both microarray datasets were quantile normalized by the RMA method [52] and similarly corrected for batch effects [54] prior to computing the proliferation and immune metagene scores. The log_2_ transformed metagene scores were then mean-centered and their distributions examined by boxplot analysis (Additional file 2). While not identical, the major features of the metagene distributions appeared largely conserved between the surgical and FNA specimens, suggesting their general comparability to one another.

### Statistical analyses

In the panel of 680 cases having tumor response data, a series of simple logistic regression models were fit examining each of the metagenes (B/P, T/NK, M/D and P) and subtype separately to determine associations with tumor response (odds ratio and 95% confidence interval) (Table 2). Metagenes were entered as continuous variables and subtype was entered as a categorical variable unless otherwise specified. We next examined each immune metagene’s association with tumor response while adjusting for proliferation and subtype (Table 2). We then stratified the data into tertiles based on the proliferation metagene and once again examined each immune metagene’s association with tumor response (Table 3). Next, we fit a stepwise logistic regression model within each tertile to see whether one (or more) metagenes were independently associated with tumor response. We then examined the association of each immune metagene with tumor response separately by each cancer subtype (basal-like, CL, HER2-enriched (HER2-E), LumB, and LumA; Table 4). In addition, we used a chi-square test to determine whether there was a relationship between treatment type and tumor response. Finally, we fit two stepwise logistic regression models to predict tumor response with 6 or 11 potential predictor variables: estrogen receptor (ER) status, the P metagene, BP, TNK, and MD metagenes and tumor subtype considered as one six-level categorical variable (Table 5) or tumor subtype considered as six individual binary variables (that is, LumA yes/no, LumB yes/no, and so on) (Table 6). Analyses were performed using SAS version 9.3 (SAS Institute Inc., Cary, NC, USA).

**Table 2:** **Logistic regression analysis for associations with tumor response, with and without adjustment for the proliferation metagene and subtype**

**Variable**	**Odds ratio**	***P*** **-value** ^**b**^	**Adjusted odds ratio** ^**c**^	***P*** **-value**
	**(95% CI)** ^**a**^		**(95% CI)**	
B/P	1.60 (1.35-1.89)	<0.0001	1.41 (1.18-1.69)	0.0002
T/NK	1.59 (1.32-2.05)	0.0004	1.66 (1.23-2.25)	0.001
M/D	1.69 (1.35-2.11)	<0.0001	1.66 (1.28-2.15)	0.0001
P	2.54 (1.90-3.41)	<0.0001	-	-
Subtype^d^	-	<0.0001	-	-

B/P = B cells/plasma B cells; CL, Clauin-Low subtype; CI = confidence interval; LumA = Luminal A; LumB = Luminal B; M/D = monocyte/dendritic cell population; T/NK = T cell/natural killer cell-specific population; P = proliferation.

^a^95% confidence interval. ^b^Likelihood ratio test *P*-value. ^c^Adjustment for P metagene as a continuous variable and subtype as a categorical variable. ^d^Odds ratios (versus normal subtype) and 95% CI and *P*-value for each subtype are: basal-like (1.33; 0.43-4.10; 0.0006), CL (1.12; 0.32-3.87; 0.13), HER2-enriched (1.08; 0.33-3.56; 0.10), LumA (0.18, 0.06-0.60; <0.0001) and LumB (0.55; 0.17-1.75; 0.16).

**Table 3:** **Univariate response analysis of metagenes stratified by proliferation tertile**

**Variable** ^**a**^	**Odds ratio**	***P*** **-value**
	**(95% CI)**	
**P** ^**L**^ **n = 222 (pr = 20, nr = 202)**		
B/P	1.96 (1.30-2.93)	0.001
T/NK	1.70 (1.04-2.77)	0.03
M/D	1.64 (1.00-2.71)	0.51
**P** ^**I**^ **n = 230 (pr = 48, nr = 182)**		
B/P	1.39 (1.06-1.83)	0.02
T/NK	2.17 (1.30-3.61)	0.003
M/D	1.95 (1.30-2.93)	0.001
**P** ^**H**^ **n = 228 (pr = 82, nr = 146)**		
B/P	1.50 (1.15-1.96)	0.003
T/NK	1.96 (1.24-3.07)	0.004
M/D	1.96 (1.37-2.82)	0.0002

B/P = B cells/plasma B cells; CI = confidence interval; M/D = monocyte/dendritic cell population; P^H^ = high proliferation tertile; P^I^ = intermediate proliferation tertile; P^L^ = low proliferation tertile; T/NK = T cell/natural killer cell-specific population.

^a^For each tertile, 'n' is the number of cases in specified proliferation tertile, 'pr' is the number of responders, and 'nr' is the number of nonresponders.

**Table 4:** **Univariate response analysis of metagenes stratified by subtype**

**Variable** ^**a**^	**Odds ratio**	***P*** **-value**
	**(95% CI)**	
**Basal-like n = 205 (pr = 72, nr = 133)**		
B/P	1.26 (0.95-1.68)	0.12
T/NK	1.53 (0.93-2.51)	0.09
M/D	1.66 (1.15-2.39)	0.007
**Claudin-low n = 47 (pr = 17, nr = 30)**		
B/P	1.92 (1.10-3.34)	0.02
T/NK	2.08 (0.80-5.47)	0.14
M/D	1.11 (0.43-2.86)	0.82
**HER2-E n = 72 (pr = 26, nr = 46)**		
B/P	1.31 (0.84-2.04)	0.23
T/NK	0.83 ( 0.36-1.92)	0.67
M/D	1.19 (0.54-2.59)	0.67
**LumB n = 133 (pr = 23, nr = 110)**		
B/P	1.33 (0.93-1.89)	0.11
T/NK	1.44 ( 0.86-2.42)	0.16
M/D	1.71(1.01-2.91)	0.05
**LumA n = 207 (pr = 7, nr = 200)**		
B/P	1.52 (0.82-2.83)	0.19
T/NK	1.16 (0.52-2.60)	0.72
M/D	0.93 (0.43-2.02)	0.85

B/P = B cells/plasma B cells; CI = confidence interval; HER2-E, HER2-enriched; LumA = Luminal A; LumB = Luminal B; M/D = monocyte/dendritic cell population; T/NK = T cell/natural killer cell-specific population.

^a^For each subtype, 'n' is the number of cases, 'pr' is the number of responders, and 'nr' is the number of nonresponders.

**Table 5:** **Stepwise model with intrinsic subtype entered as categorical variable**

**Variable**	**Odds ratio**	***P*** **-value**
**(n = 662)**	**(95% CI)**	
P	2.28 (1.64-3.18)	<0.0001
ER status^a^	2.14 (1.45-3.18)	0.0002
M/D	1.49 (1.12-1.99)	0.0065
B/P	1.24 (1.01-1.54)	0.045

B/P = B cells/plasma B cells; ER = estrogen receptor; M/D = monocyte/dendritic cell population; P = proliferation.

^a^Binary variable (negative versus positive).

**Table 6:** **Stepwise model with intrinsic subtype entered as individual variables**

**Variable**	**Odds ratio**	***P*** **-value**
**(n = 662)**	**(95% CI)**	
M/D	1.64 (1.28-2.11)	<0.0001
P	1.97 (1.36-2.85)	0.0003
ER status^a^	1.90 (1.26-2.88)	0.002
LumA^a^	1.94 (1.03-3.66)	0.04

ER = estrogen receptor; LumA = Luminal A; M/D = monocyte/dendritic cell population; P = proliferation.

^a^Binary variable (negative versus positive).

## Results

We assembled a microarray database of gene expression profiles of breast tumor biopsies from a multicenter meta-cohort of 701 breast tumor patients who received neoadjuvant chemotherapy (Table 1). From this database, we re-constructed the three immune metagenes (B/P, T/NK and M/D) and a proliferation (P) metagene as previously described [36] and as outlined in the Methods section. Briefly, a metagene is defined as a cluster of coordinately expressed gene transcripts whose expression levels, within a tumor, can be averaged to generate a single metagene score that reflects the composite transcriptional activity level of the gene cluster [32,36]. In Nagalla *et al.* [36] we found that these scores (for each of the three immune metagenes) exhibited prognostic value by Cox regression analysis, with high metagene scores associated with prolonged patient DMFS. The prognostic value, however, was largely restricted to the highly proliferative tumors defined by the upper tertile of the proliferation metagene scores (that is, the most proliferative tumors).

To determine the therapy-predictive value of the immune metagene scores in the neoadjuvant setting, we used simple (that is, single explanatory variable) logistic regression models to examine each metagene, individually, followed by multiple (that is, multiple explanatory variables) logistic regression to measure associations between immune metagenes, the P metagene, tumor subtype and tumor response to chemotherapy. Measures of tumor response were based on previously assigned scores of RCB (0 = complete pathologic response, 1 = minimal residual disease, 2 = moderate residual disease and 3 = extensive residual disease) or determination of the presence or absence of a clinical pCR. Tumors with RCB scores of 0 or 1, or that achieved a pCR (in the absence of assigned RCB scores) were coded as '1' to designate a positive response; all other instances were coded as '0' to designate a negative response. In the group of 680 patients annotated for tumor response, univariate analyses revealed highly significant associations between tumor response and all five covariates (the immune metagenes, the P metagene and intrinsic subtype) with high immune and P metagene scores, and basal-like subtype, being associated positively with tumor response, and LumA subtype being associated with negative tumor response (Table 2).

Next, we investigated the dependence of the therapy-predictive performance of the immune metagenes on tumor proliferative capacity and molecular subtype. First, we examined the association of each metagene with tumor response while adjusting for the proliferation metagene and subtype. As shown in Table 2, the adjusted odds ratios for each immune metagene remained highly significant, indicating that each metagene contributes additive predictive information independent of proliferation and subtype, and is not simply recapitulating information about tumor response already conveyed by those variables. To examine this more closely, we next stratified cases into proliferation (P) tertiles (low (P^L^), intermediate (P^I^) and high (P^H^)) and the association of each immune metagene with tumor response was examined as a function of P tertile (Table 3). Significant positive associations were observed for all immune metagenes within each of the three P tertiles, with the exception of the M/D metagene in the P^L^ tertile. We then fit three stepwise multiple logistic regression models, one for each P tertile, to determine whether or not multiple metagenes would retain significance in a single model. We found that only one immune metagene achieved significance in each P tertile. In the P^L^ tertile, only the B/P metagene remained significant (*P* = 0.001), while only the M/D metagene remained significant in the P^I^ and P^H^ tertiles (*P* = 0.001 and *P* = 0.0002, respectively). This result reflects the degree of collinearity between the three immune metagenes, particularly M/D and T/NK, which have a Spearman correlation of 0.80 (Additional file 3). We next investigated the predictive value of the metagenes in the context of the intrinsic molecular subtypes, as we previously observed the prognostic value of the metagenes to segregate most significantly with the basal, HER2-E and LumB subtypes [36]. As shown in Table 4, the M/D and B/P metagenes achieved statistical significance in certain subtypes, despite potential limitations owing to variability in sample size. While all three metagenes trended towards significant positive associations with tumor response in the basal and LumB subtypes, only the M/D metagene achieved a significant association in these two subtypes. By contrast, the B/P metagene achieved significance in the CL subtype despite small sample size. None of the metagenes displayed a significant association within the HER2-E and LumA subtypes.

Based on reports that indicate an immuno-modulatory role for anthracyclines and taxanes [59–62], we investigated the possible impact of exposure to these drugs in relation to tumor response. Using a chi-square test, we examined a 4 × 2 table (treatment by response) to see whether a relationship existed between treatment type and tumor response in this meta-cohort. No statistically significant association was observed. Furthermore, treatment type did not mediate the observed associations between individual metagenes and tumor response (data not shown).

To better understand the predictive value of the immune metagenes in the presence of other clinical and predictive covariates, we fit multiple logistic regression models on the 662 cases with complete annotation for the variables listed below. Specifically, we fit two stepwise logistic regression models to predict tumor response using either 6 or 11 potential predictor variables: ER status, P, B/P, TN/K, and M/D metagenes, and tumor subtype considered as one six-level categorical variable (Table 5) or tumor subtype considered as six individual binary variables (Table 6). The goal of this analysis was to identify a subset of variables that retained a significant association with tumor response when included together in the logistic regression model. When subtype was considered as one categorical variable, we found that ER status, P, M/D, and B/P metagenes all were retained in the model as statistically significant predictors of tumor response (*P* <0.05). However, when we re-fit the model with subtypes entered as individual binary variables, we found that the LumA subtype was added as a statistically significant predictor, along with ER status, P and M/D metagenes. In this model, B/P was no longer statistically significant (*P* >0.05) and was not selected for inclusion by the stepwise procedure. Despite these differences between the two stepwise logistic regression models, both showed a strong and consistent association of ER status, P and M/D metagenes (*P* <0.007 for all variables) with tumor response, suggesting that these variables each explain different aspects of tumor response to neoadjuvant chemotherapy.

## Discussion

Over the past decade, a number of tumor expression profiling studies have identified transcriptomic signatures unique to tumor-infiltrating immune cells. Often revealed by hierarchical clustering techniques or outcome correlation studies, these signatures distinguish multiple different immune cell types [32–37] and recapitulate immunohistochemistry-based observations in breast cancer that link tumor-infiltrating immune cell abundance to disease-free survival and overall survival of patients [30,36–42]. More recently, similar studies involving pre-surgical breast tumor biopsies have begun to demonstrate associations between immunity-related genes and tumor responsiveness to neoadjuvant chemotherapy [22,44,45,63,64]. However, a unified understanding of how immunity-related genes relate to both patient prognosis and therapy prediction has yet to be addressed. In Nagalla *et al*. [36], we recently reported the discovery of three immune metagenes with highly significant and independent associations with patient DMFS; however, the significance of these associations was found to depend largely on tumor proliferative capacity and intrinsic molecular subtype. In the current study, we hypothesized that the same immune metagenes may exhibit similarly significant associations with tumor response to neoadjuvant chemotherapy. To test this hypothesis, we analyzed a collection of 701 microarray expression profiles of primary breast tumor biopsies and corresponding clinical data, including tumor responsiveness to neoadjuvant chemotherapy. Overall, we found that each of the immune metagenes, B/P, T/NK and M/D, was highly significantly and positively associated with tumor response (Table 2), thereby confirming the duality of their roles as biomarkers of favorable outcome in both patient prognosis and therapy prediction.

However, certain biological variables that influence the strength of these associations were found to vary between the prognostic and therapy-predictive settings. Whereas the prognostic performance of the immune metagenes observed in Nagalla *et al.* [36] was found to be mostly restricted to highly proliferative tumors (P^H^), this was not the case for their therapy-predictive performance. With the exception of the M/D metagene, which did not reach significance in the P^L^ tertile, all metagenes were found to be significantly associated with positive tumor response in each of the proliferation tertiles and with similar odds ratios (Table 3). In multivariate analysis, we observed mostly collinear relationships among the metagenes, with only single metagenes retaining significance in each proliferation tertile. Specifically, only the B/P metagene remained significant in the P^L^ tertile, while only the M/D metagene remained significant in the P^I^ and P^H^ tertiles. Taken together, these observations suggest that tumor proliferative capacity may modify the prognostic and therapy-predictive potentials of the immune metagenes in fundamentally different ways. While the prognostic attributes of the immune metagenes (but not their therapy-predictive attributes) exhibit dependency on a high proliferative capacity, the predominating therapy-predictive power of the immune metagenes may vary from one immune compartment to another in a proliferation-dependent manner (for example, B/P versus M/D).

With respect to intrinsic molecular subtypes, the therapy-predictive associations could not be resolved to the same degree as that of the prognostic associations observed in Nagalla *et al.* due to smaller sample sizes that prevented simultaneous stratification by both subtype and proliferation tertile. However, stratification by subtype alone did reveal several interesting therapy-predictive associations (Table 4). First, the majority of odds ratios trended toward positive and significant tumor responses in three subtypes - basal-like, LumB and CL - while showing few to no associations in the HER2-E and LumA subtypes. Similarly, in Nagalla *et al.*, we found that the immune metagenes were strongly associated with favorable DMFS in the majority of basal-like and LumB tumors, but not LumA tumors. By contrast, however, in Nagalla *et al.* we observed that the immune metagenes were not associated with prognosis in CL tumors, not as a whole, nor when partitioned into proliferation tertiles. Interestingly, these findings may indicate that CL tumors exemplify a condition wherein the prognostic and therapy-predictive roles of the immune metagenes diverge, rather than parallel one another. However, given the paucity of CL tumors represented in our microarray dataset, this hypothesis warrants further investigation in larger sample populations. Another discordant observation pertained to the HER2-E tumor subtype. While the immune metagenes were prognostic of DMFS in the majority of HER2-E tumors analyzed (that is, the P^H^ population), we did not observe a parallel association with tumor response in HER2-E tumors in the current study. Whether this observation would hold true in the P^H^ subpopulation of HER2-E tumors could not be determined due to limiting sample size.

Taken together, these observations indicate that while the immune metagenes are associated with both patient prognosis and chemotherapy response, the tumor properties that influence these associations (proliferation and subtype) are not consistent in their effects. A plausible explanation may relate to the anti-tumor biology mirrored by the immune metagenes and the impact of chemotherapy on tumor immunogenicity. As surrogate markers of immune cell abundance, and by virtue of their positive associations with both DMFS and chemotherapy response, the immune metagenes appear to reflect the anti-tumor potential of the host immune system. Neoadjuvant chemotherapy is known to impact tumor-specific immune responses in various ways. Central among these is the ability of chemotherapy to enhance tumor-specific immunogenicity. For example, chemotherapy-induced cell death can trigger the release of tumor-associated antigens or cell death-associated molecules leading to a cascade of anti-tumor immune responses that can contribute to tumor regression [16,65,66]. Alternatively, chemotherapeutic agents are known to exert a variety of other immunostimulatory effects, including: 1) induction of MHC class I expression and subsequent presentation of tumor antigens [67]; 2) increased expression of ligands that stimulate tumor-reactive activation of NK and cytotoxic T cells [68,69]; 3) induction of tumor-expressing death receptors responsive to ligands expressed by immune effectors [70]; and 4) the depletion or inactivation of tumor-protective regulatory T cells [71,72]. In the neoadjuvant setting, such mechanisms of chemotherapy-induced immunogenicity may operate independent of tumor proliferation rate and intrinsic molecular subtype. Thus, while it remains uncertain as to why the prognostic power of the immune metagenes is restricted to highly proliferative breast tumors and certain molecular subtypes [36], their therapy-predictive power may be influenced more by the prevalence of chemotherapy-induced immunogenic mechanisms than by tumor phenotypes that dictate immunogenic potential in the absence of neoadjuvant treatment.

As our study involved the comparison of gene expression metrics between prognostic and therapy-predictive settings, an underlying assumption was that microarray expression profiles are comparable between different types of tissue biopsies - namely surgical tumor biopsies (SURGbx) obtained at tumor resection (that is, the basis for our previous prognostic observations) and fine-needle aspirate biopsies (FNAbx) or core biopsies (Cbx) obtained prior to surgery (the basis for our current therapy-predictive observations). Previously, Symmans and colleagues compared the cellular compositions and expression profiles between breast FNAbx and Cbx [58]. They reported similar proportions of immune cell infiltrates (on average, 15% (FNAbx) and 20% (Cbx)) but discordant proportions of malignant epithelial cells (80% (FNAbx) versus 50% (Cbx)) and stromal cells (5% (FNAbx) versus 30% (Cbx)). While unsupervised hierarchical clustering revealed a high degree of gene correlation between patient-matched FNA and core biopsies overall, disproportionate cell compositions among biopsy types were shown to result in skewed distributions for certain cell type-specific gene expression patterns. Whether this is true for SURGbx and FNAbx has, to our knowledge, not been investigated. The proliferation and immune metagenes examined in our current study derived predominantly from expression profiles of FNAbx (confirmed for 69% of samples (n = 482)) but also included a smaller unspecified number of Cbx intermixed with FNAbx (31% of samples). In Nagalla *et al*. [36] we defined and characterized the metagenes based solely on SURGbx specimens (n = 1,954). Thus, we used this opportunity to compare and contrast metagene score distributions between the SURGbx and FNAbx microarray datasets by box and whisker plot analysis (Additional file 2). In each metagene comparison, both the interquartile ranges (boxes) and the spreads among the lower and upper quartiles (whiskers) showed good concordance between FNAbx and SURGbx datasets, suggesting that the distributions of the proliferation and immune metagenes are fairly comparable among these different biopsy types. Nevertheless, a more rigorous investigation of the impact of biopsy method on gene expression dynamics would be warranted for clinical diagnostic applications.

To date, the published data on immune gene signatures predictive of breast tumor response to neoadjuvant chemotherapy derives from four studies that have focused mainly on genes implicated in the biology of tumor infiltrating lymphocytes [22,44,45,64]. Surprisingly, we observed very little overlap between these four published gene sets and our immune metagenes. The greatest overlap was seen with our T/NK metagene. Of the 46 genes comprising our T/NK metagene, 12 are included in one or more of the published gene sets, with at least two T/NK genes overlapping with each of the four gene sets. By contrast, however, only one of the 40 genes comprising our B/P metagene, and one of the 19 genes comprising our M/D metagene showed overlap with a published gene set. Thus, we conclude that the therapy-predictive attributes of the B/P and MD metagenes are mostly unexplored, representing novel biomarkers of breast tumor response to neoadjuvant chemotherapy.

By stepwise logistic regression, we discerned that the M/D and B/P metagenes gave the most robust therapy-predictive performances among the immune metagenes. In a similar vein, both the B/P and M/D metagenes were strong and independent predictors of DMFS in Nagalla *et al.*, whereby the B/P metagene emerged as the most significant immune covariate by multivariate analysis. In the context of therapy prediction, however, the M/D metagene prevailed as the most significant and additive immunity-related covariate in the final multivariate model.

The genes comprising the M/D metagene are overexpressed in myeloid cell lineages and enriched for functions associated with antigen processing and presentation [36]. Of the 19 genes comprising the M/D metagene, nine are involved in MHC class II-mediated antigen presentation (*HLA-DRA*, *HLA-DRB1*, *HLA-DMA*, *HLA-DMB*, *HLA-DPA1*, *HLA-DPB1*, *HLA-DQA1*, *HLA-DQB1*, *CD74*), suggesting that antigen presentation may constitute the driving biology behind the metagene’s therapy-predictive power. While B cells also express MHC class II molecules, the M/D metagene is absent of B cell markers but inclusive of *CSF1R*, which encodes the classical macrophage colony-stimulating factor receptor that controls growth and differentiation of macrophages and dendritic cells [73]. Thus, the biology underlying the M/D metagene is consistent with a myeloid-driven, anti-tumor immune response engendered by either macrophages (for example, M1-polarized) or dendritic cells functionally oriented towards tumor rejection. Dendritic cells (DCs) are professional antigen presenting cells that coordinate innate and adaptive immune responses to cancer. Dying tumor cells, such as those succumbing to chemotherapy-induced apoptosis, emit danger signals interpreted by DCs as damage-associated molecular patterns (DAMPs) [74]. These signals can induce DC maturation, production of pro-inflammatory cytokines, tumor cell engulfment and subsequent processing and presentation of tumor antigens [15,75,76]. Moreover, anthracyclines and taxanes can stimulate DC-mediated antigen presentation either indirectly, through induction of a DC-responsive immunogenic form of tumor cell death [16], or directly, through DC exposure to chemotherapy (at low or noncytotoxic concentrations) resulting in the up-regulation of MHC and co-stimulatory molecules on the surface of DCs [77]. These observations, and in light of the prognostic and therapy-predictive attributes of the M/D metagene described herein, suggest the possibility that DC-based cancer vaccines [78] could synergize with conventional breast cancer chemotherapeutics, and if administered in the neoadjuvant setting, could prime a durable immunogenic response that not only contributes to primary tumor regression but provides protection against recurrent disease.

## Conclusion

Our findings demonstrate the existence of distinct transcriptional footprints of infiltrating effector immune cell subpopulations in breast tumors that are predictive of both chemotherapeutic efficacy and reduced risk of metastatic recurrence. From a biological perspective, these metagenes underscore the important participation of different arms of the immune system in the chemotherapy-induced rejection of established breast tumors as well as the prevention of distant recurrence in the presence or absence of adjuvant treatment [36]. Furthermore, our work indicates that antigen presentation may play a more prominent role in the efficacy of neoadjuvant chemotherapy of breast cancer than previously recognized, and may explain, in part, the variability of pathologic response in the neoadjuvant setting. As reporters of immunogenic potential, the immune metagenes could have functionality as actionable therapeutic markers, particularly in this era of expanding immunotherapies. How the immune metagenes might be harnessed to inform clinical decisions early in the therapeutic sequence warrants further investigation.

## Additional files

Additional file 1:
**Patient and clinical characteristics of MDACC-701.**
Additional file 2:
**Comparison of metagene distributions by biopsy type.** Mean-centered metagene scores derived from microarray expression profiles of surgical samples (SURGbx; n = 1,954) and fine-needle aspirate biopsies (FNAbx; n = 482) were compared by box and whisker plot analysis. The upper and lower bounds of each box delineate the interquartile range (25th to 75th percentiles) and the horizontal black line within the box designates the median of the distribution. Upper and lower whiskers (T-bars) demarcate the 95th and 5th percentiles, respectively. Open circles mark the outliers (below and above the 5th and 95th percentiles, respectively). Red and green dashed lines mark the upper and lower tertile thresholds. Additional file 3:
**Collinearity between the three immune metagenes.**

## Abbreviations

B/P: B cells/plasma B cells
Cbx: core biopsy
CL: claudin-low
DC: dendritic cell
DMFS: distant metastasis-free survival
ER: estrogen receptor
FNA: fine needle aspirate
FNAbx: fine-needle aspirate biopsy
GEO: Gene Expression Omnibus
HER2-E: HER2-enriched
LumA: luminal A
LumB: luminal B
M/D: monocyte/dendritic cell population
MDACC: MD Anderson Cancer Center
MHC: Major histocompatibility complex
P: Proliferation
pCR: complete pathologic response
P^H^: high proliferation tertile
P^I^: intermediate proliferation tertile
P^L^: low proliferation tertile
RCB: Residual cancer burden
RMA: Robust Multi-array Average
SURGbx: Surgical tumor biopsy
T/NK: T cell/natural killer cell-specific population
