# Supplementary figures and images for: Dual roles for immune metagenes in breast cancer prognosis and therapy prediction
Source: Genome Med. 2014 Oct 28;6(10):80. doi: 10.1186/s13073-014-0080-8 (PMC4240891; doi:10.1186/s13073-014-0080-8)

Additional File 2

5


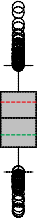

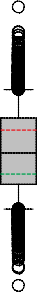

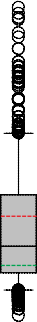

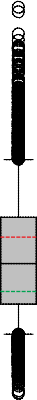

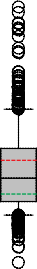

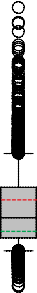

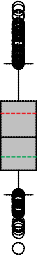

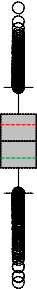


4

3

2

metagene score

1

0

-1

-2

-3

Supplement: Additional file 2: — Comparison of metagene distributions by biopsy type. Mean-centered metagene scores derived from microarray expression profiles of surgical samples (SURGbx; n = 1,954) and fine-needle aspirate biopsies (FNAbx; n = 482) were compared by box and whisker plot analysis. The upper and lower bounds of each box delineate the interquartile range (25th to 75th percentiles) and the horizontal black line within the box designates the median of the distribution. Upper and lower whiskers (T-bars) demarcate the 95th and 5th percentiles, respectively. Open circles mark the outliers (below and above the 5th and 95th percentiles, respectively). Red and green dashed lines mark the upper and lower tertile thresholds. [file 13073_2014_80_MOESM2_ESM.docx]
