# Supplementary material for: Dual roles for immune metagenes in breast cancer prognosis and therapy prediction
Source: Genome Med. 2014 Oct 28;6(10):80. doi: 10.1186/s13073-014-0080-8 (PMC4240891; doi:10.1186/s13073-014-0080-8)
Supplement: Additional file 3: — Collinearity between the three immune metagenes. [file 13073_2014_80_MOESM3_ESM.docx]

|  | **P** | **B/P** | **TN/K** | **M/D** |
| --- | --- | --- | --- | --- |
| **P** | 1.0 | 0.05 | -0.17 | -0.11 |
| **B/P** | 0.05 | 1.0 | 0.55 | 0.56 |
| **TN/K** | -0.17 | 0.55 | 1.0 | 0.80 |
| **M/D** | -0.11 | 0.56 | 0.80 | 1.0 |

**Additional File 3. Table showing pair-wise Spearman correlations between metagenes in the MDACC-701 cohort.**
